# MRgFUS Pallidothalamic Tractotomy for Chronic Therapy-Resistant Parkinson's Disease in 51 Consecutive Patients: Single Center Experience

**DOI:** 10.3389/fsurg.2019.00076

**Published:** 2020-01-14

**Authors:** Marc N. Gallay, David Moser, Franziska Rossi, Anouk E. Magara, Maja Strasser, Robert Bühler, Milek Kowalski, Payam Pourtehrani, Christian Dragalina, Christian Federau, Daniel Jeanmonod

**Affiliations:** ^1^SoniModul, Center for Ultrasound Functional Neurosurgery, Solothurn, Switzerland; ^2^Praxisgemeinschaft für Neurologie, Bern, Switzerland; ^3^Neurologische Praxis Solothurn, Solothurn, Switzerland; ^4^Neurological Division, Bürgerspital Solothurn, Solothurn, Switzerland; ^5^Privatklinik Obach, Solothurn, Switzerland; ^6^Rodiag Diagnostics Centers, Solothurn, Switzerland; ^7^Eden Reha- und Kurklinik, Oberried am Brienzersee, Switzerland; ^8^Department of Radiology, University Hospital Basel, Basel, Switzerland; ^9^Institute for Biomedical Engineering, ETH Zürich, University Zürich, Zurich, Switzerland

**Keywords:** pallidothalamic tractotomy, functional stereotactic neurosurgery, minimally invasive, high intensity MR-guided focused ultrasound, Parkinson's disease

## Abstract

**Background:** There is a long history, beginning in the 1940s, of ablative neurosurgery on the pallidal efferent fibers to treat patients suffering from Parkinson's disease (PD). Since the early 1990s, we undertook a re-actualization of the approach to the subthalamic region, and proposed, on a histological basis, to target specifically the pallidothalamic tract at the level of Forel's field H1. This intervention, the pallidothalamic tractotomy (PTT), has been performed since 2011 using the MR-guided focused ultrasound (MRgFUS) technique. A reappraisal of the histology of the pallidothalamic tract was combined recently with an optimization of our lesioning strategy using thermal dose control.

**Objective:** This study was aimed at demonstrating the efficacy and risk profile of MRgFUS PTT against chronic therapy-resistant PD.

**Methods:** This consecutive case series reflects our current treatment routine and was collected between 2017 and 2018. Fifty-two interventions in 47 patients were included. Fifteen patients received bilateral PTT. The median follow-up was 12 months.

**Results:** The Unified Parkinson's Disease Rating Scale (UPDRS) off-medication postoperative score was compared to the baseline on-medication score and revealed percentage reductions of the mean of 84% for tremor, 70% for rigidity, and 73% for distal hypobradykinesia, all values given for the treated side. Axial items (for voice, trunk and gait) were not significantly improved. PTT achieved 100% suppression of on-medication dyskinesias as well as reduction in pain (*p* < 0.001), dystonia (*p* < 0.001) and REM sleep disorders (*p* < 0.01). Reduction of the mean L-Dopa intake was 55%. Patients reported an 88% mean tremor relief and 82% mean global symptom relief on the operated side and 69% mean global symptom improvement for the whole body. There was no significant change of cognitive functions. The small group of bilateral PTTs at 1 year follow-up shows similar results as compared to unilateral PTTs but does not allow to draw firm conclusions at this point.

**Conclusion:** MRgFUS PTT was shown to be a safe and effective intervention for PD patients, addressing all symptoms, with varying effectiveness. We discuss the need to integrate the preoperative state of the thalamocortical network as well as the psycho-emotional dimension.

## Introduction

Stereotactic neurosurgery for Parkinson's disease (PD) has a long history, beginning in the 1940s, with targets placed on different sections of the pallidothalamic fiber system. Meyers proved in the early 1940s that basal ganglia surgery could be done without impairing consciousness (1, 2). After ablations of anterior parts of the striatum, he moved to ansotomies (3), using first mechanical lesioning and later an early high intensity focused ultrasound system (4–7). Targeting pallidal efferent fibers was further developed and refined by others (8–18). Bilateral lesional approaches have been intensively explored in the sixties mostly (10, 19–24) and have shown highly variable side-effects, the description of which often lacked precision in term of intensity. In addition, older techniques were less precise and much larger lesions were performed, with sometimes extensive thalamic damage and risk of capsular encroachement. In the early 1990s, deep brain stimulation came to dominate the field, setting aside in most centers further refinements and developments in ablative stereotaxy. Nowadays, in the context of improved technologies, bilateral interventions are being considered to deserve further studies (25).

In the early 1990s, we undertook an update of the subthalamic approach (26, 27), later supported by a three-dimensional histological description of the pallidothalamic fiber system in the subthalamus (28). We proposed to target specifically the pallidothalamic tract just below the thalamus at the level of Forel's field H1, where the ansa lenticularis and fasciculus lenticularis converge. We named this intervention pallidothalamic tractotomy (PTT), a more precise denomination than “campotomy,” as Forel described three fiber fields in the subthalamus named “Haubenfascikeln” H, H1 and H2. The PTT has been performed during 20 years with radiofrequency (26) and since 2011 with the MR-guided focused ultrasound (MRgFUS) technique (29). PTT corresponds to an optimized pallidotomy, because it allows, with very limited tissue ablation, an extensive liberation of the thalamocortical dynamics from pallidal overinhibition (see below), while leaving the thalamus intact. A prospective open-label study and a case report describing similar subthalamic approaches have been recently published (30, 31).

The limited current interest for fiber tract ablations in the subthalamus (but not for chronic stimulation procedures) may be explained (1) by uncertainties concerning the anatomical course of the pallido- and cerebellothalamic tracts (28, 32) and (2) by the necessity to hit a small fiber bundle (and not a larger nuclear, pallidal or thalamic, area) closely surrounded by relevant structures, i.e., the mammillothalamic tract (MTT), the subthalamic nucleus, the internal capsule (IC) and the somatosensory thalamus (12, 33). Such topographic conditions require a targeting precision inside the millimeter, which the MRgFUS technology can provide (29, 34).

There are pathophysiological arguments to choose pallidal efferent fibers among other targets in the treatment of medically refractory chronic PD (26, 27, 29, 35). In Parkinson, a chain reaction is triggered by the loss of dopamine producing cells in the substantia nigra (36–40). It results in increase and decrease of cell activities in the striatum followed by an overactivity of the internal pallidum, which chronically overinhibits the pallidal-recipient thalamic relay cells in the thalamus (27, 41). This overinhibition has been proposed to be at the source of the initiation and maintenance of a thalamocortical dysrhythmic process (35, 42–45), measurable thanks to quantitative EEG (46, 47) and responsible for the production of the parkinsonian symptoms.

Nowadays, different approaches using MRgFUS are applied in PD, either targeting the motor thalamus, the internal pallidum or the subthalamic nucleus (48–52).

Our first experience with MRgFUS PTT was collected by repeating sonications on the same spot (29). This led to the appearance of partial therapeutic effects in the follow-up of some patients. A next step has been to use longer application times. This approach was not sufficient and required further developments. A recent reappraisal of the histological anatomy of the pallidothalamic tract in H1 was combined with an optimization of our lesioning strategy using thermal dose control, with the goal to refine target coverage and thus provide a further step in the prevention of recurrences or partial therapeutic effects (53, 54). We hereby present the clinical results in PD of the first consecutive 56 uni- or bilateral MRgFUS PTTs applying this new approach.

## Methods

### Ethics

All patients treated with this protocol signed an informed consent form after having been fully informed about the treatment, its results and risks. No ethical approval was sought because MRgFUS PTT has been approved by the Federal Office of Public Health (FOPH) of Switzerland and is covered by swiss social insurances.

### Study Context

Patients were referred to receive specifically MRgFUS interventions. There was no patient randomization and no blinding. This single center consecutive and prospective case series reflects our current treatment routine of chronic therapy-resistant PD with the MRgFUS PTT. It was collected between January 2017 and September 2018. Monitoring for primary outcomes as well as cognitive and adverse event evaluations were performed by senior independent neurologists 3 months and 1 year after the procedure, in the context of a swiss federal registry on functional neurosurgical MRgFUS interventions. All available data of this consecutive series were analyzed cross-sectionally in December 2018, thus explaining variable “*n*” values given in Results, as not all patients reached the 1 year follow-up at this moment.

### Selection Criteria

Selection criteria for MRgFUS PTT included (1) idiopathic PD (diagnosed by neurologists), (2) chronic disease with at least 1 year of therapy resistance, characterized by (a) insufficient efficacy of L-Dopa dosed up to at least 600 mg L-Dopa equivalents per day, with symptom control during maximum 50% of the day, (b) gastro-intestinal or other side-effects, (c) fluctuations (on-off phenomenon), and (d) on-medication dyskinesias (choreoathetosis), (3) intensity of symptoms (intensity of tremor at rest and/or hypobradykinesia of 3/4 or more), (4) strongly diminished quality of life, and (5) Montreal Cognitive Assessment (MoCA) test in the norm or reduced but not below 20/30. Three subtypes of idiopathic PD were considered (55): the tremor dominant (TD), the akineto-rigid (AR) and the mixed form (MX). Asymmetry of symptoms was not a selection criterion. No prefixed age limit was set. All patients were examined by a senior internist for any medical contraindications. Antiaggregant therapy was stopped for 10 days before the intervention, and normal coagulation and blood pressure were checked for all patients prior to surgery.

### Procedure

The procedures were performed in a 3T MR imaging system (GE Discovery 750, GE Healthcare, Milwaukee, WI, USA) using the ExAblate Neuro device (InSightec, Haifa, Israel). Targeting was performed using the stereotactic multiarchitectonic Morel Atlas of the Human Thalamus and Basal Ganglia (56) and its developments (28, 53). Three-dimensional stereotactic coordinates were measured on the MR images, based on the intercommisural line and the thalamo-ventricular border. The center of the PTT target was located 6.5 mm from the medial thalamic border (L 6.5), 1 mm posterior to the MCL (MCL-1) and on the intercommissural plane. Sonications had the shortest possible time and the corresponding power in order to provide a thermal dose of 240 CEM at each focal point which represents a conservative value corresponding to a 100% probability of lesion in a volume of 1.5 × 1.5 × 3.0 mm. The detailed technical description of the targeting and realization of the MRgFUS PTT using thermal dose control has been published in a separate work (53).

**Figure 1:**
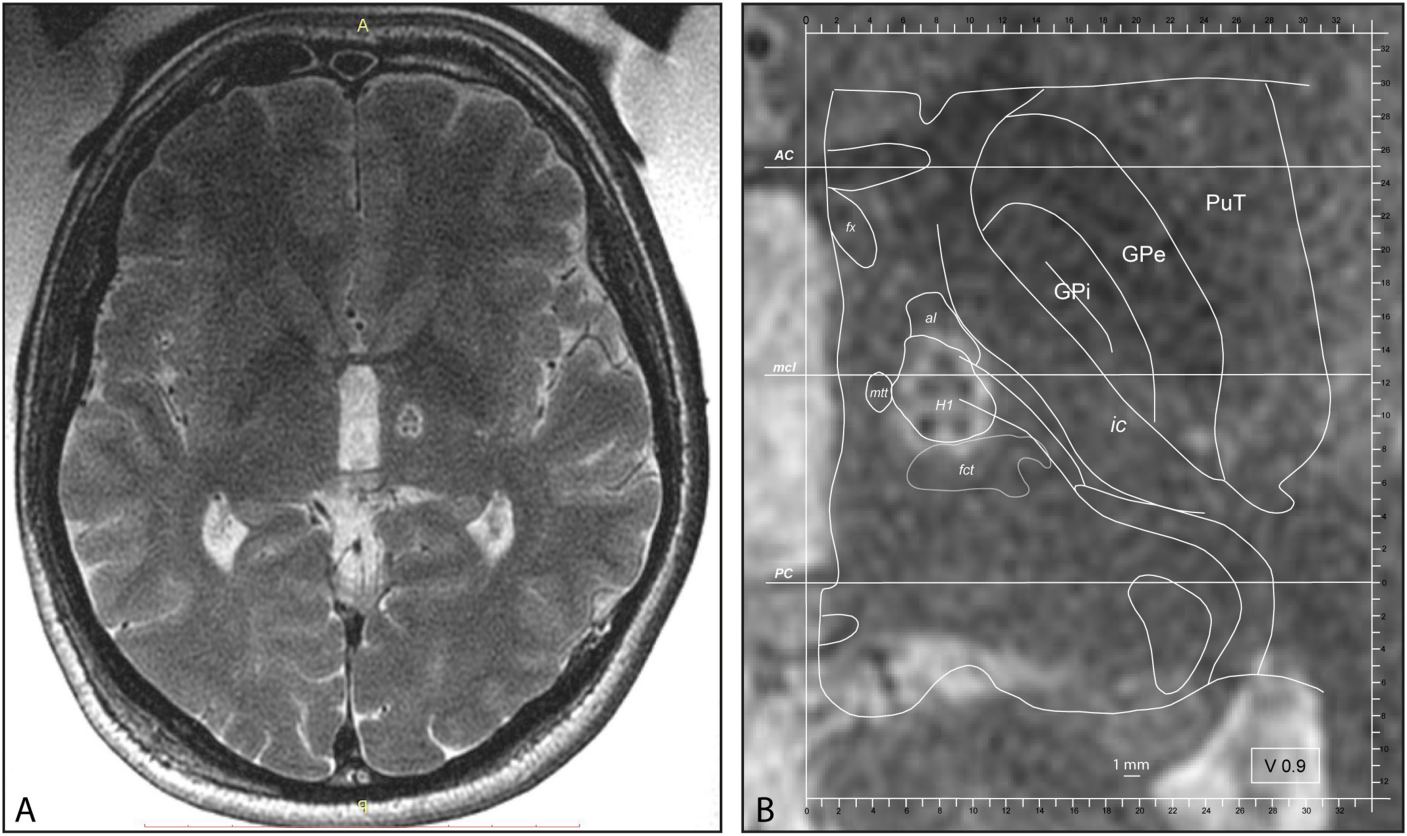
**(A)** Intraoperative MR axial T2 scan with body coil after PTT lesion, cut through the intercommissural plane. **(B)** PTT at higher magnification taken intraoperatively with superimposed atlas maps 0.9 mm ventral to the intercommissural plane, modified from the Morel Atlas of the Human Thalamus and Basal Ganglia. AC, anterior commissure; al, ansa lenticularis; fct, fasciculus cerebello-thalamicus; fx, Fornix; GPe, globus pallidus, external segment; GPi, globus pallidus, internal segment; H1, H1 field of Forel; ic, internal capsule; mcl, midcommissural line; mtt, mammillothalamic tract; PC, posterior commissure; PuT, putamen.

Two patients had a contralateral centrum medianum thalamotomy (CMT) in addition to PTT during the same session. Mean maximal sonication power was 1,000 ± 265 [W] (range 500–1,400 W) using the shortest possible sonication durations. MR imaging was performed for co-registration preoperatively and 2 days after the treatment. Figure 1 shows a PTT target as seen at the end of the procedure. Target reconstruction was performed for every patient according to Moser et al. (29, 34, 57, 58). All interventions took place in an ambulatory setting with one night stay at a nearby clinic for comfort. In two patients (3.6%) hospitalization was longer than one night, in one case for comfort and in the second because of tiredness and pre-existent mobility issues. No patient of this series had to be acutely hospitalized during the first postoperative month except for pre-planned physical rehabilitation programs.

As to bilateral interventions, the second one took place at least 6 months after the first PTT. It was performed if the symptomatology on the untreated side was strong and therapy-resistant enough (with an intake between 300 and 600 mg L-Dopa equivalents per day) to justify surgery. Complementation of first PTTs performed before this series (with sonication repetition or long sonication times) was coupled with the treatment of the second side during the same session except in one case.

Baseline preoperative L-Dopa equivalent intake (range of intake between 0 and 1,400 mg/day, not taking into account dopamine agonists) corresponded to the intake during the last weeks prior to treatment and not to the maximum tried dosages along the whole disease history before the intervention. In patients showing small up to no effect to L-Dopa or having suffered from side-effects, it was indeed stopped usually long before the intervention period. The reduction in L-Dopa intake was supervised by the referring neurologist. It was undertaken according to the wish and readiness of patients to perform it.

### Clinical Evaluation and Outcome Measures

The primary endpoints at 3 months and 1 year postoperatively were (1) the Unified Parkinson's Disease Rating Scale (UPDRS) scores in on and off states (59, 60), (2) assessment by the patient her/himself of the global symptom relief for the treated body side (GSRt), of the tremor control on the treated side and of the global symptom relief for the entire body (GSRb), (3) reduction in drug intake, (4) off dystonia, (5) on dyskinesias (choreo-athetosis), (6) sleep disturbances, (7) pain, and (8) adverse events.

As to secondary endpoints, the MoCA test (61) was performed in the few months up to 2 days prior to the treatment, and repeated 2 days and 1 year after it. In addition, all patients were asked to fill the questionnaire for the activities of daily living according to Bain et al. (62), the World Health Organization Quality of Life (WHOQOL bref) (63), and the Hospital Anxiety and Depression Scale (HADS) (64) preoperatively, 3 months and 1 year after the treatment.

Follow-up assessments took place 2 days, 3 months, and 1 year after the procedure. On- and off-medication examinations were performed preoperatively as well as at 1 year. At 2 days and 3 months patients were usually seen in an on-state. For international patients, telemedicine (video and phone conversation) were offered for both postoperative evaluations when unable to travel long distances.

### Statistics

Statistical analysis of quantitative scores compared with baseline was carried out by repeated ANOVA measures and multiple comparisons were applied using a *post-hoc* analysis with Bonferroni-Holm testing (Daniel's XL toolbox; https://www.xltoolbox.net/). Statistical significance was set to α < 0.05.

## Results

**Figure 2:**
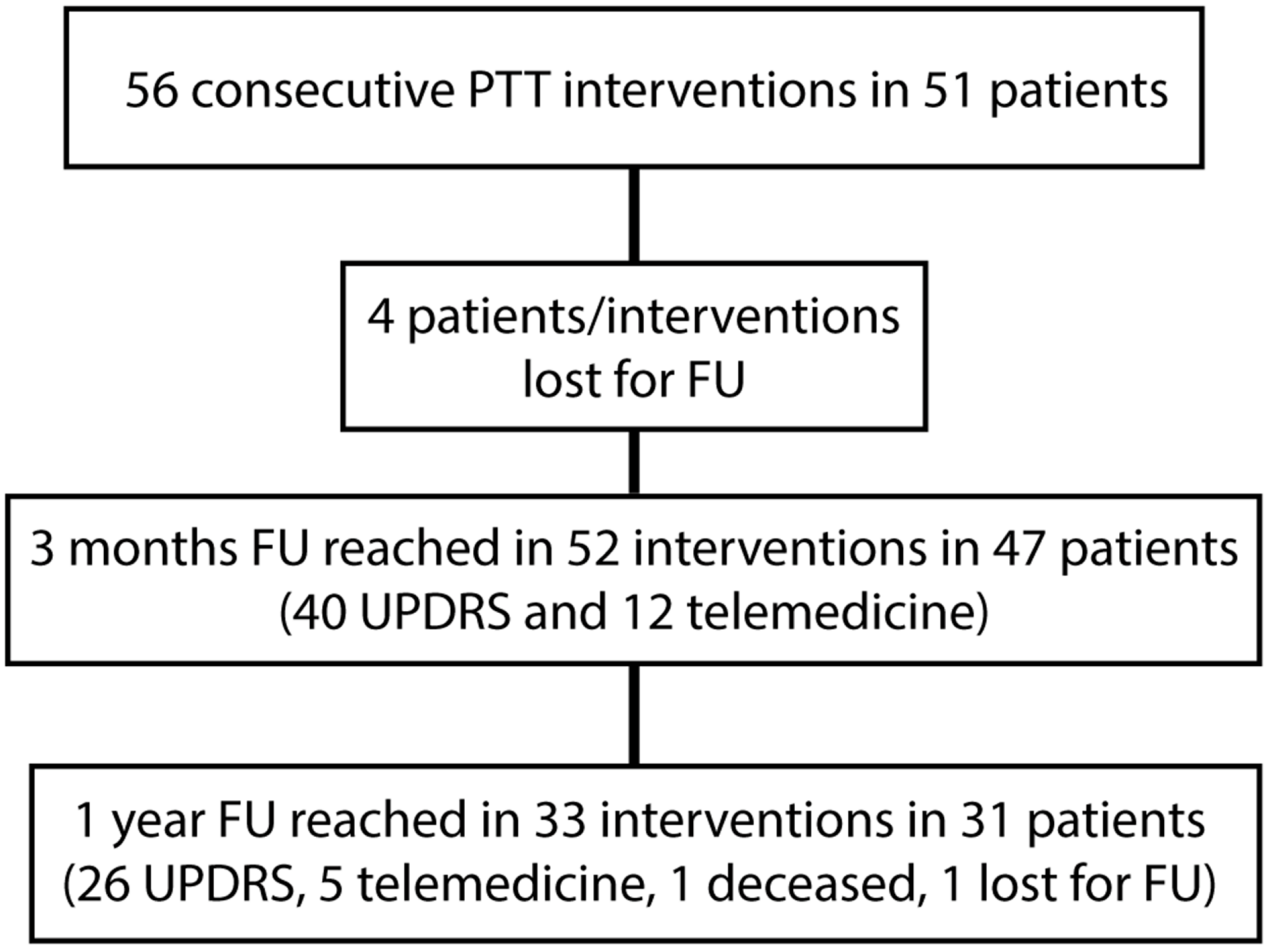
Flow diagram showing follow-ups (FU) of this cross-sectional study. Telemedicine stands for video and phone conversation instead of regular neurological examinations for patients unable to travel long distances.

Patient characteristics are summarized in Table 1. Fifty-two of 56 (92%) interventions in 47 patients were included in this study. Four patients were lost to follow up (8%). All of them were international patients who could be reached by phone 3 months after the treatment but did not provide at least a video examination. The “*n*” values in the tables stand for “interventions” as some patients were treated in two sessions. The 3 months follow-up was reached in 52 interventions (47 patients) and the 1 year follow-up in 33 interventions (31 patients) in December 2018 (see Figure 2). At 1 year, one patient was deceased and one was lost for FU. UPDRS total scores were available for all interventions at baseline, the UPDRS III on-medication was missing in two patients and the UPDRS III off-medication in three patients. At 1 year, 26 total UPDRS scores were collected and five patients were interviewed with telemedicine. The variability in “*n*” values in Table 2 is due to the inclusion of clinical data obtained through telemedicine. UPDRS III on-medication scores were missing in five patients and off-medication scores in two patients.

**Table 1:** Patients characteristics.

Consecutive patients	51
Interventions	56
Age at operation [mean ± SD (min; max)] (years)	67.3 ± 10.1 (32; 88)
Age at diagnosis [mean ± SD (min; max)] (years)	57.8 ± 9.9 (30; 77)
Symptom duration [mean ± SD (min; max)] (years)	10.0 ± 5.3 (2; 26)
Males	37 (72.5%)
Ethnicity	48 Caucasians, 2 Hindus, 1 Chinese
Follow-up (months) [mean ± SD (min; max)]	9.8 ± 5.8 (3; 21)
Median follow-up time (months)	12
Tremor-dominant PD (TD)	9 (18%)
Mixed PD (MX)	37 (72%)
Akineto-rigid PD (AR)	5 (10%)
Unilateral interventions	41
Bilateral PTT	15
PTT left	35
PTT right	32
Centrum medianum thalamotomy (CMT)	2
Total targets	69
2 targets in one session	13 (11 bilateral PTT, 2 CMT)
Targets complements	10 PTT
Hoehn and Yahr stage [mean ± SD (min; max)]	2.6 ± 0.7 (1; 4)

**Table 2:** Changes from baseline to 3 months and 1 year postoperative.

**52 interventions considered for the results**	**Baseline**	***n***	**3 months**	***n***	***p***	**1 year postoperative**	***n***	***p***
Total UPDRS, on-medication (/195)	58 ± 19	48	36 ± 19	32	<0.001	32 ± 20	21	<0.001
Total UPDRS, off-medication (/195)	65 ± 20	49	39 ± 17	19	<0.001	32 ± 21	25	<0.001
UPDRS I (/16)	3.9 ± 2.1	52	2.6 ± 2.1	40	<0.01	2.4 ± 1.9	26	0.1
UPDRS II (/52)	16.1 ± 5.5	52	10.9 ± 5.2	40	<0.001	9.0 ± 5.8	26	<0.001
Speech (item 5) (/4)	1.3 ± 1.0	52	1.1 ± 0.8	40	0.15	1.0 ± 0.9	26	0.88
Total UPDRS III on-medication (/104)	34 ± 15	50	21 ± 14	31	<0.01	18 ± 12	21	<0.001
Operated side, UPDRS III, on-medication (/36)^*^	15.6 ± 7.2	50	4.9 ± 3.4	33	<0.001	3.3 ± 2.8	21	<0.001
Less affected side, UPDRS III, on-medication (/36)^*^	9.2 ± 7.0	50	9.1 ± 7.4	33	0.95	9.1 ± 8.1	21	0.99
Tremor, operated side on-medication (/12)^†^	4.7 ± 3.4	50	1.0 ± 1.7	35	<0.001	0.6 ± 1.0	24	<0.001
Rigidity, operated side on-medication (/8)^§^	3.3 ± 2.0	50	0.7 ± 1.2	33	<0.001	0.8 ± 1.1	21	<0.001
Distal hypobradykinesia operated side on-medication (/16)^¶^	7.7 ± 4.0	50	2.8 ± 2.5	33	<0.001	1.5 ± 1.9	21	<0.001
Speech on-medication (/4) (item 18)	1.1 ± 0.8	50	0.8 ± 0.8	34	0.14	0.7 ± 0.6	21	0.41
Axial items UDPRS III on-medication (/32)^#^	8.9 ± 4.6	50	7.9 ± 4.7	33	0.2	5.8 ± 3.8	21	0.28
Total UPDRS III off-medication (/104)	40 ± 15	49	21 ± 11	20	<0.001	18 ± 13	24	<0.001
Operated side, UPDRS III, off-medication (/36)^*^	19.2 ± 5.8	49	4.8 ± 3.7	19	<0.001	3.7 ± 3.3	24	<0.001
Less affected side, UPDRS III, off-medication (/36)^*^	10.8 ± 7.7	49	9.5 ± 6.5	19	0.63	9.5 ± 8.6	24	0.52
Tremor, operated side off-medication(/12)^†^	6.0 ± 3.1	49	1.1 ± 1.5	19	<0.001	0.8 ± 1.2	25	<0.001
Rigidity, operated side off-medication (/8)^§^	3.9 ± 1.7	49	1.1 ± 1.5	19	<0.001	0.9 ± 1.3	24	<0.001
Distal hypobradykinesia operated side off-medication (/16)^¶^	9.3 ± 3.6	49	2.6 ± 2.4	19	<0.001	2.0 ± 2.0	24	<0.001
Speech off medication (/4) (item 18)	1.2 ± 0.8	49	1.1 ± 0.6	19	0.82	0.8 ± 0.7	24	0.13
Axial items UDPRS III off-medication (/32)^#^	9.8 ± 4.9	49	7.9 ± 4.2	19	0.34	5.8 ± 3.8	24	<0.04
Presence of Choreo-athetosis on operated side	38%	52	2%	41	<0.001	0%	26	<0.001
Presence of Dystonia on operated side	67%	52	24%	41	<0.001	8%	25	<0.001
Sleep disturbances	52%	52	17%	41	<0.001	16%	25	<0.01
Pain on operated side	73%	52	22%	41	<0.001	16%	25	<0.001
Mean L-Dopa (mg L-Dopa equivalent) intake (range), median L-Dopa equivalent	613 ± 342 (0; 1,400), median: 600 mg	52	380 ± 340 (0; 1,350) (42% mean reduction), median: 300 mg	44	<0.01	227 ± 307 (0; 1,000) (55% mean reduction), median: 75 mg	28	<0.01
Dopaminagonists intake	17 (33%)	52	7 (14%)	49	0.05	3 (10%)	29	0.17

*For UPDRS scores, higher values indicate stronger impairments. P-values were calculated using post-hoc multiple-comparisons with baseline. “n” stand for number of interventions included in the analysis*.

* *For the operated side: UPDRS III items 20.1, 20.3, 21.1, 22.2, 22.4, 23.1, 24.1, 25.1, 26.1 or 20.2, 20.4, 21.2, 22.3, 22.5, 23.2, 24.2, 25.2, 26.2*.

† *UPDRS III items 20.1, 20.3, 21.1 or 20.2, 20.4, 21.2*.

§ *UPDRS III items 22.2, 22.4 or 22.3, 22.5*.

¶ *UPDRS III items 23.1, 24.1, 25.1, 26.1 or 23.2, 24.2, 25.2, 26.2*.

# *UPDRS III items 18, 19, 22, 27, 28, 29, 30, 31*.

Mean age at the time of the treatment was 67 ± 10 years. Mean symptom duration was 10.0 ± 5.3 years. The mean and median follow-up times were 9.8 ± 5.8 and 12 months, respectively. Mean Hoehn and Yahr stage was 2.6 ± 0.7. Of the three subtypes of idiopathic PD, 72% were MX, 18% TD and 10% AR. Forty-one over 56 interventions were unilateral. Fifteen patients received bilateral PTT either in one session (*n* = 2) or staged. PTT target complementation in patients operated before this series was performed in 10 cases.

Mean skull density ratio (SDR) was 0.57 ± 0.11 (median: 0.57, range: 0.31–0.77). SDR ratio below 0.3 was an exclusion criterion. One patient only was denied treatment during the duration of this study because of a low SDR value. This SDR distribution was in accordance with our previous experience with a population dominantly Caucasian.

### Primary Outcome Measures

**Figure 3:**
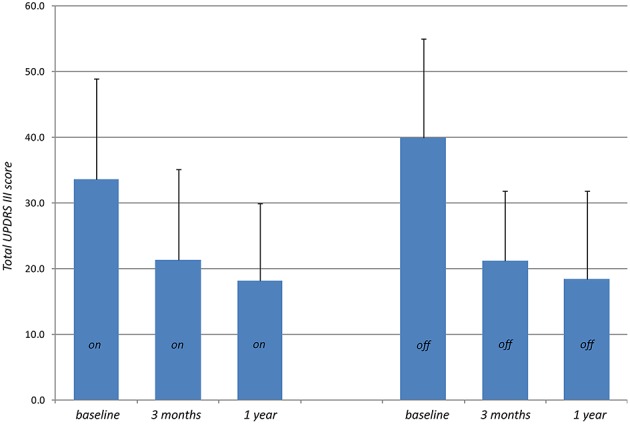
Total UPDRS III scores (higher values indicate stronger impairments) measured preoperatively (baseline) on-medication and 3 months and 1 year after PTT off-medication.

**Figure 4:**
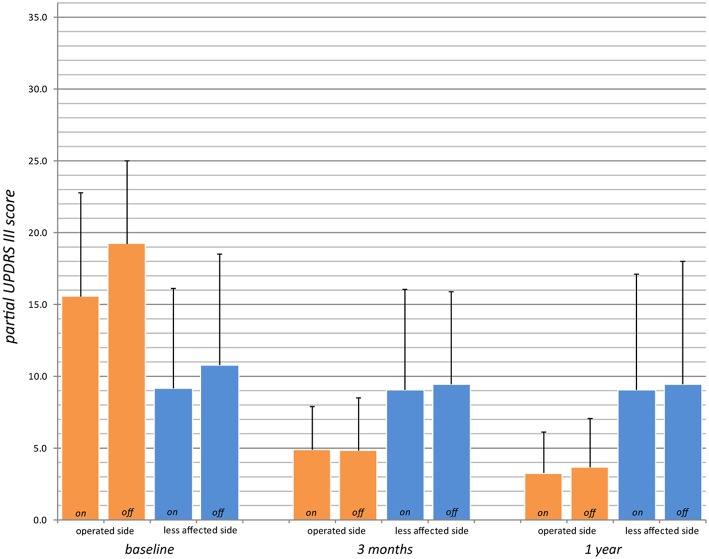
Partial UPDRS III scores for the operated side (UPDRS III items 20.1, 20.3, 21.1, 22.2, 22.4, 23.1, 24.1, 25.1, 26.1 or 20.2, 20.4, 21.2, 22.3, 22.5, 23.2, 24.2, 25.2, 26.2; max. 36 points, higher values indicate stronger impairments) preoperatively (baseline), at 3 months and 1 year in on- and off-medication state (for *n* values, see Table 2).

Primary outcome measures are presented in Tables 2, 3. Reduction of the mean total UPDRS score was 38% (*p* < 0.001) at 3 months and 46% (*p* < 0.001) at 1 year on-medication, 41% (*p* < 0.001) and 51% (*p* < 0.001) off-medication, respectively. Mean UPDRS I was reduced at 3 months (33%, *p* < 0.01) and 1 year (38%, *p* = 0.1). Mean UPDRS II score reductions reached statistical significance at 3 months (32%, *p* < 0.001) and 1 year (44%, *p* < 0.001). Mean score for Speech (item 5 in UPDRS II) was slightly improved but this was not statistically significant (*p* = 0.15 at 3 months and *p* = 0.88 at 1 year). Reduction of the total UPDRS III score was 37% (*p* < 0.01) at 3 months and 46% (*p* < 0.001) at 1 year on-medication, and 47% (*p* < 0.001) and 54% (*p* < 0.001) off-medication, respectively (Figure 3). Mean of the UPDRS III items related only to the most affected, and thus treated side (items 20.1, 20.3, 21.1, 22.2, 22.4, 23.1, 24.1, 25.1, 26.1 or 20.2, 20.4, 21.2, 22.3, 22.5, 23.2, 24.2, 25.2, 26.2) was reduced at 3 months (69%, *p* < 0.001) and 1 year (79%, *p* < 0.001) in on-medication, 75% (*p* < 0.001) and 81% (*p* < 0.001) in off-medication state, respectively. Mean UPDRSIII for the un-operated side did not change significantly at 1 year follow-up (Figure 4).

**Table 3:** One year post PTT off-medication vs. preoperative on-medication.

	**Percentage reduction of the mean at 1 year *off-* vs. preoperative *on-*medication**	***n***	***p***
Tremor (/12)^*^	84% (0.8 ± 1.2 vs. 5.2 ± 4.0), 83.3% improved, 8.3% stable and 8.3% increased	24	<0.001
Rigidity (/8)^†^	70% (0.9 ± 1.3 vs. 2.9 ± 2.0), 88% improved and 12% unchanged	24	<0.001
Distal hypobradykinesia (/16)^§^	73% (2.0 ± 2.0 vs. 7.3 ± 3.9), 96% improved and 4% unchanged	24	<0.001
Axial items (/32)^¶^	24% (6.0 ± 4.1 vs. 7.8 ± 4.0), 67% improved, 12% stable, 21% worsened	24	0.13
Speech (/4) (UPDRS III item 18)	38% (0.5 ± 0.7 vs. 0.8 ± 0.7), 46% improved, 42% stable, 13% worsened	24	0.17

* *Depending on the operated side: UPDRS III items 20.1, 20.3, 21.1 or 20.2, 20.4, 21.2. n stands for number of interventions having reached 1 follow-up*.

† *UPDRS III items 22.2, 22.4 or 22.3, 22.5*.

§ *UPDRS III items 23.1, 24.1, 25.1, 26.1 or 23.2, 24.2, 25.2, 26.2*.

¶ *UPDRS III items 18, 19, 22, 27, 28, 29, 30, 31*.

**Figure 5:**
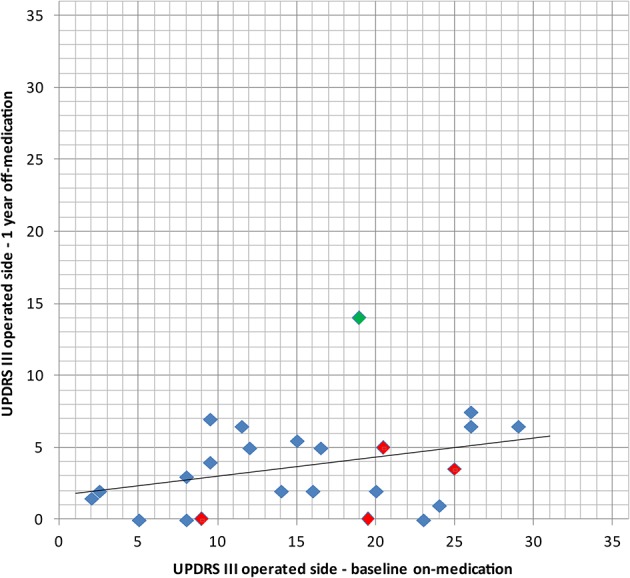
Comparison of the baseline UPDRS III for the operated side in on-medication state with the 1 year postoperative examination in off-medication state with its linear regression line, thus comparing medication vs. surgery alone. In red: bilaterally treated patients. In green: patient with subjective full recurrence.

**Figure 6:**
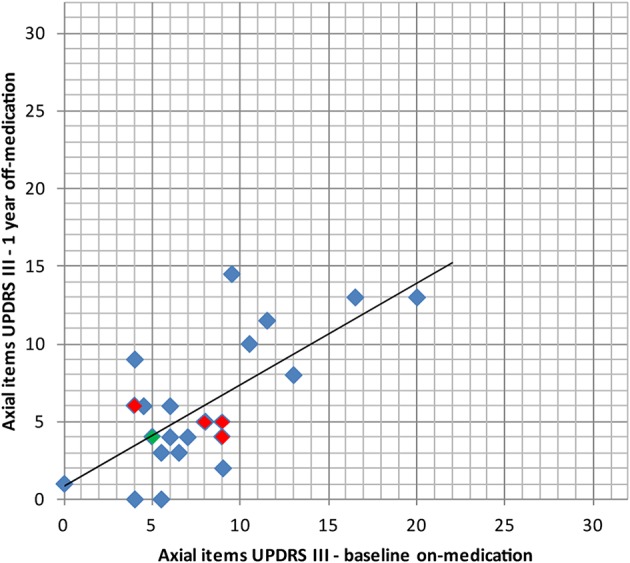
Comparison of the axial items of the UPDRS III (items 18, 19, 22, 27, 28, 29, 30, 31, higher values indicating stronger impairments, max. 32 points) preoperatively (baseline) in on-medication state and 1 year after PTT in off-medication state with its linear regression line. In red: bilaterally treated patients. In green: patient with subjective full recurrence.

Percentage reduction of the mean of the off-medication postoperative (1 year) vs. on-medication preoperative scores (Table 3 and Figure 5) was calculated for tremor component (UPDRS III items 20.1, 20.3, and 21.1 or 20.2, 20.4, and 21.2), rigidity (items 22.2 and 22.4 or 22.3 and 22.5), distal hypobradykinesia (items 23.1, 24.1, 25.1, and 26.1 or 23.2, 24.2, 25.2, and 26.2), axial items (18, 19, 22, and 27–31) and speech (item 18). It was 84% (*n* = 24, *p* < 0.001) for tremor, 70% (*n* = 24, *p* < 0.001) for rigidity and 73% (*n* = 24, *p* < 0.001) for distal hypobradykinesia. For axial items (Figure 6), it was 24% (*n* = 24, *p* = 0.13), with 67% of patients improved and 21% worsened. For voice, it was 38% (*n* = 24, *p* = 0.17) with 46% of patients improved and 13% worsened. There was a worsening of more than three points in two patients for axial items and no worsening more than one point for speech.

Off-medication dystonia was present on the dominant side in 67% (*n* = 35/52) of the preoperative examinations. It was found in 24% (*p* < 0.001, *n* = 10/41) at 3 months and in 8% (*p* < 0.001, *n* = 2/25) only at 1 year. Thirty-eight percent (*n* = 20/52) of cases displayed on-medication dyskinesias for the dominant side preoperatively; it was 2% (*p* < 0.001, *n* = 1/41) at 3 months and none (*p* < 0.001, *n* = 0/25) at 1 year. REM sleep was disturbed in 52% of patients preoperatively (n = 27/52), 17% (*p* < 0.001, *n* = 7/41) at 3 months and 16% (*p* < 0.01, *n* = 4/25) at 1 year. Parkinson associated pain in the operated body side concerned 73% (*n* = 38/52) of patients prior to treatment. At 3 months they were 22% (*p* < 0.001, *n* = 9/41) and 16% (*p* < 0.001, *n* = 4/25) at 1 year.

### Drug Intake Reduction

Preoperative mean L-Dopa equivalent intake was 613 ± 342 mg (median: 600 mg), 380 ± 340 mg (median: 300 mg, range: 0–1,350 mg) at 3 months (42% mean reduction, *p* < 0.01), and 227 ± 307 mg (median: 75 mg, range: 0–1000) at 1 year (55% mean reduction, *p* < 0.01). Thirty-three percent of patients took dopamine agonists (pramipexol, ropinirol, or rotigotin). They were 14% (*p* = 0.05) 3 months after and 10% (*p* = 0.17) at 1 year.

### Subjective Assessments

Subjective assessments are presented in Table 4. Mean percentage of tremor relief was 87 ± 13% (*n* = 40, median: 90%, 100% improved ≥ 50%) at 3 months and 88 ± 19% (*n* = 29, median: 95%, 97% improved by ≥ 70%) at 1 year. One patient did not see any improvement in his tremor at 1 year. The GSRt at 3 months was 75 ± 24% (*n* = 37, median: 80%, 92% improved ≥ 50%) and 82 ± 22 (*n* = 29, median: 90%, 93% improved by ≥ 50%) at 1 year. The GSRb at 3 months was 64 ± 26% (*n* = 40, median: 70%, 83% improved ≥ 50%) and 69 ± 27 (*n* = 27, median: 80%, 85% improved by ≥ 50%) at 1 year.

**Table 4:** Subjective outcome measures rated by the patients.

	**3 months**	***n***	**1 year postoperative**	***n***
Subjective tremor relief (%)^*^	87 ± 13 (median: 90%) (100% improved by ≥50%)	40	88 ± 19 (median: 95%) (97% improved by ≥70%) (3% not improved, *n* = 1)	29
Subjective global symptom relief in treated side (GSRt) (%)	75 ± 24 (median: 80%) (92% improved by ≥50%)	37	82 ± 22 (median: 90%) (93% improved by ≥50%)	29
Subjective global symptom relief for both sides (GSRb) (%)	64 ± 26 (median: 70%) (83% improved by ≥50%)	40	69 ± 27 (median: 80%) (85% improved by ≥50%)	27

* *Akineto-rigid subtype not included. n stands for number of interventions included in the analysis*.

### Secondary Outcome Measures

Secondary outcome measures are presented in Table 5. MoCA mean scores did not change from baseline [27.2 ± 2.3, range (21–30), *n* = 56] either at 2 days [27.3 ± 2.9, range (16–30), *n* = 56] or at 1 year [27.6 ± 2.9, range (18–30), *n* = 25] after the procedure. At 2 days MoCA was decreased more than two points after six interventions in five patients. The maximal score reduction was from 21/30 to 16/30. Twenty-eight percent remained unchanged and 43% improved their score at 2 days. At 1 year, of the six significant score reductions, three recovered completely (two over three were even improved by two points from baseline) and three have not yet reached the 1 year follow-up. Only one patient, aged 88, suffered a significant MoCA reduction along the year after the intervention, sometime between the two follow-ups. Bilateral PTT subgroup analysis showed no changes in MoCA score 2 days after the second side (27.6 ± 3.5 at 2 days vs. 27.5 ± 2.7 preoperatively, *n* = 15).

**Table 5:** Secondary outcome measures from baseline (2 days, 3 months, and 1 year after treatment).

	**Baseline**	***n***	**2 days**	***n***	***p***	**1 year**	***n***	***p***
MoCA [mean ± SD (min, max)]	27.2 ± 2.3 (21, 30)	56	27.3 ± 2.9 (16, 30)	56	0.85	27.6 ± 2.9 (18, 30)	25	0.69
			**3 months**					
HADS [mean ± SD (min, max)]^*^	14.3 ± 6.7 (1, 34)	52	12.4 ± 8.7 (0, 40)	43	0.2	10.4 ± 6.9 (0, 27)	26	0.1, *p* < 0.02 for Anxiety
WHOQOL bref preoperative^†^	90 ± 14 (53, 121)	52	96 ± 13 (69, 123)	43	<0.03	99 ± 17 (69, 126)	24	0.4
WHOQOL-Bref item 1 (how would you rate your quality of life)^§^	2.9 ± 1 (1, 5)	52	3.4 ± 1 (1, 5)	43	0.002	3.6 ± 1 (2, 5)	24	0.08
WHOQOL-Bref item 2 (How satisfied are you with your health?)^¶^	2.3 ± 1 (1, 4)	52	3.3 ± 1 (1, 5)	43	<0.001	3.4 ± 1 (1, 5)	24	0.002
WHOQOL-Bref item 17 (How satisfied are you with your ability to perform your daily living activities?)^¶^	3.1 ± 1 (1, 5)	52	3.4 ± 1 (1, 5)	43	0.09	3.4 ± 1 (1, 5)	24	0.7
Activities of daily living (62)^#^	46.1 ± 12 (26, 85)	51	40.7 ± 13 (25, 87)	44	0.07	39.7 ± 11.6 (25, 63)	26	0.1

*n stands for number of interventions included in the analysis*.

* *(0–42): high scores indicating more severe anxiety and depression levels*.

† *Possible range 26-130: high scores indicating better quality of life*.

§ *1–5 (1: very poor, 2: poor, 3: neither poor nor good, 4: good, 5: very good)*.

¶ *1–5 (1: very dissatisfied, 2: dissatisfied, 3: neither satisfied nor dissatisfied, 4: satisfied, 5: very satisfied)*.

# *(100–25): higher scores indicating stronger impairments*.

Hospital Anxiety and Depression Scale (HADS), The World Health Organization Quality of Life questionnaire (WHOQOL bref) and Activities of daily living questionnaire showed improvements of mean scores at 3 months and 1 year. Statistical significance was only reached for WHOQOL item 2 (How satisfied are you with your health?) at 3 months (*p* < 0.001) and 1 year (*p* < 0.005) and for WHOQOL item 1 (how would you rate your quality of life) at 3 months (*p* = 0.002).

### Postoperative Dynamics

Six patients (11%) showed fluctuations of consciousness (increased sleepiness up to confusional states) which all resolved completely within 24 h. One patient displayed a short-lived (<12 h) fluctuating corticospinal syndrome (paresis with Babinski sign) without evidence of capsular involvement according to the applied thermal doses as well as to the intraoperative post-lesion MR-examination.

### Adverse Events

There was no bleeding, no infection and no ballism. Sonications were painful for a few seconds in seven patients (13%). No patient reported lasting significant headache >6 h after the procedure. One patient developed a scalp hypoesthesia around one pin fixation which had fully recovered after 3 months. One patient suffered from a short-lived intense anxio-depressive episode from which he rapidly and completely recovered, with no relapse till more than 1 year postoperatively. At 3 months follow-up, seven patients reported increased (two patients) or new (five patients) speech difficulties (UPDRS II, item 5), at 1 year they were 2 and 4, respectively. In these six cases, hypophonia was increased by one point in half of them and two points in the other half. An objective increase in hypophonia (UPDRS III, item 18) at 1 year in off-medication state (compared with preoperative off-medication state) was present in three patients (by one point only) over 24. Out of the four patients among them treated bilaterally and controlled at 1 year, one was improved, one unchanged and the last two worsened by one point. There was no clear-cut dysarthria in the whole series. One patient was seen in an outpatient clinic for an L-Dopa over-dosage. One patient suffered from a hiccup as well as difficulties for breathing and speech lasting over months but regressive at 10 months. One patient had a fall 1 month after surgery and broke his hip. He underwent hip surgery and was walking without aid at 3 months follow-up. One patient died 5 months after PTT from a gastrointestinal occlusion.

## Discussion

This prospective case series of 56 consecutive interventions for PD performed in 2017 and 2018 is placed in the context of an 8-year long experience with the MRgFUS PTT against PD. Symptom recurrences or partial symptom control led us to develop a target protocol to optimize target coverage, including (1) a histological reappraisal of the antero-posterior extension of the pallidothalamic tract taking into account interindividual variability, and (2) the use of preplanned focal point displacement, shortest sonication duration and thermal dose control. Indeed, neither repetition of sonications nor prolonging sonication duration on the same focal point brought sufficient consistency in patient symptom control (53). This evolution integrates itself in a learning curve development: for example, the tremor relief on the operated side was estimated by the patients to be 52% at the beginning of our experience, moving up to 60% when we repeated sonications on the same spot [see Magara et al. (29)], 70% when we increased the sonication times, and finally 88% currently, as described in this study.

Patients reported an 88% mean (95% median) tremor, 82% mean (90% median) global symptom relief on the operated side and 69% mean (80% median) global symptom relief for the whole body at 1 year. These results compare well with our scale improvement ratings: at 1 year the off-medication state was compared with the baseline on-medication state and revealed a percentage reduction of the mean of 84% for tremor, 70% for rigidity, and 73% for peripheral hypobradykinesia. Patients' reports on their own surgical outcomes cover their whole daily living time. They provide thus a useful complement to the clinical snapshots collected during the short time frame of control examinations (49, 65).

PTT achieved suppression of on-medication dyskinesias on the operated side in all patients as well as strong reduction in pain, dystonia and sleep problems. Reduction of L-Dopa intake was performed slowly and respecting patient readiness. Reduction of the mean L-Dopa intake at 1 year was 55% with a median intake of 75 mg L-Dopa equivalent/day. Dopamine agonists were reduced more readily and were only taken by three patients after 1 year.

The most resistant symptoms in this series were speech and axial symptoms, as they did not improve significantly at 1 year follow-up, although the trend was toward improvement for both. No capsular or thalamic dysarthria was recorded. Increase of hypophonia along subjective and objective estimations concerned a limited number of patients and was slight in intensity. Although, the number of patients who received bilateral PTT and have been controlled at 1 year is small (*n* = 4), we observed similar results for this group compared to the whole patient group (*n* = 24) for axial items (see Figure 6) and speech. The bilateral PTT group is too small to allow firm conclusions at this point.

The whole and the bilateral PTT patient groups showed a stable mean MoCA score 2 days after surgery. MoCA reductions at 2 days can be attributed first to postoperative fatigue and second to reductions of thalamocortical reserves (34) in the absence of capsular or MTT encroachments. A valuable way to explore the presence or not of hemispheric significant distributed cell losses is to couple MR imaging (analysis of hemispheric atrophy) with the analysis of cognitive functions, as paralimbic/multimodal areas are very extensive and widespread throughout both brain hemispheres. We have some evidence (not quantified) that postoperative cognitive reductions correlate with the magnitude of preoperative thalamocortical atrophy. The presence of postoperative cognitive reductions in a given study will therefore depend on the limit set by the neurosurgical team as to what are acceptable preoperative cognitive deficits.

In the single patient of this series having experienced symptom recurred, re-analysis of the intervention showed partial coverage of the pallidothalamic tract due to technical difficulties. He reported a 50% symptom relief on the operated side at 3 months, but most of his symptoms had reappeared at 1 year. Figures 5, 6 shows that for this patient (green dot) some clinical benefit was nevertheless obtained. This underlines the central importance of optimal thermal dose coverage of the target (53, 54).

Two patients had a CMT on the contralateral side to PTT. This adjunction did not influence most results, as they were collected on the side treated by PTT. However, a discrete effect only on the total UPDRS cannot be excluded. There is current interest for the CMT (66, 67) in the treatment of movement disorders. It is being explored in our center as a secondary target to PTT for bilateral interventions.

In a context of brain surgery and particularly of lesioning procedures, an intervention should in our opinion provide better results than drug therapy, thus justifying surgical risks. Hence our central selection criterion of therapy resistance. As shown in Figure 4 and Table 2, postsurgical status is indeed better than preoperative medication status. We are aware that this is at variance from criteria of DBS studies (68, 69). To follow our line of thought, we have compared in this study postoperative off-medication state with preoperative on-medication state, allowing us to assess directly the superior relief obtained by surgery as compared to medical treatment.

### Pathophysiological Considerations

In view of the pathophysiological data discussed elsewhere and summarized in the Introduction, the PTT provides the possibility to liberate the thalamocortical network from pallidal overinhibition while keeping the thalamus unoperated. In our opinion, PTT is an optimized form of pallidotomy, because (1) it provides a maximal amount of suppression of overinhibiting pallidal output to the thalamus in a much smaller volume, (2) it avoids the lenticulostriatal domain, thus reducing the vascular risk of pallidal surgery, and (3) the surgical risk for the optic tract is suppressed and reduced for the internal capsule. This allows symptom relief without reduction of the thalamocortical dynamics, which is essential for our whole sensory, motor and complex mental functions. The absence of predictable and unavoidable deficits in our study after the interruption of the pallidothalamic tract confirms this statement. In our opinion, the pallidothalamic tract may be considered as an ideal target because (1) it is in position to disturb the thalamocortical dynamics, and (2) pallidal overinhibition of the thalamus may have made this pathway useless. Preexisting normal pallidothalamic functions may have been taken over by the powerful corticothalamic network, in the context of extensive redundancy and plastic abilities of the whole thalamocortical system (70). Because the PTT interruption lies upstream of the thalamus, it gives the possibility for the whole thalamocortical system to normalize, thus allowing in principle all symptoms to recede. This process needs variable amounts of time to be implemented, in view of the functional complexity of interacting thalamic and cortical partners. Clinical observation shows indeed progression in symptom controls, which are variable from symptom to symptom. The tremor for example needs an average of 2–3 months to progressively disappear, as tremor waves become shorter in duration and lower in amplitude and frequency (26).

The short but sometimes intense transient phenomena observed postoperatively (see Results/Postoperative Dynamics) correlate inversely with the amount of thalamocortical reserves (34) and are thought to be related to a physiological rebound caused by the readjustment of the thalamocortical network, as it is abruptly liberated from the incoming pallidothalamic overinhibition (26).

### The Paralimbic/Multimodal Psycho-Emotional Dimension

After PTT, improvements of axial functions were limited and hypophonia increased in some patients. This voice worsening was present in 13% of patients but never more than one UPDRS point in intensity. According to peroperative data and postoperative target reconstructions, it cannot be explained by an encroachment of the treatment zone on the internal capsule or on the thalamus. The explanation currently offered is that this would be the sign of a further unchallenged disease progression. We tend to look rather in the direction of psycho-emotional factors. Indeed, gait and voice functions are complex and bilateral, and are at the base of two essential human functions: independence and communication. The voice is surely the most sensitive of the two, having a small effector serving minute movements, and being tightly driven by emotions. The main if not exclusive speech symptom we observed is hypophonia, a symptom also often seen in depressive patients. We consider the emotional load of neurodegeneration set on the shoulders of parkinsonian patients as huge. In view of the presence of rich interconnections between the paralimbic/multimodal and motor systems, particularly but not exclusively at the level of the cingulate motor areas (71, 72), a significant effect of ideas and emotions on motricity should be considered. Such a strong psychomotor effect could well be at the source of the difficulties arising typically after the intervention for the second side, in a moment where the patients wonder intensely how things will proceed now that the surgical treatment is finished. The tensions inside the emotional network can lead to psychomotor abnormal outputs mainly in the domain of axial, voice and gait hypobradykinesia, opening the way to misinterpretations of a psychomotor effect for a sign of disease progression.

### Limitations of This Study

This consecutive surgical case series presents the obvious limitation of not being sham-controlled. Patients were not all followed for 1 year because the study was a cross-sectional one with a stop in December 2018. This provides irregular final follow-ups for the different patients. The small group of bilateral PTTs at 1 year follow-up (four patients) shows similar results as compared to unilateral PTTs but does not allow to infer firm conclusions at this point concerning the feasibility and risk profile of the bilateral PTT.

## Conclusion

This case series, which will require longer follow-up and more patients receiving bilateral treatment, supports the PTT as a safe and highly effective surgical option in the treatment of chronic therapy-resistant PD. We compared patient's on-medication preoperatively with their off-medication postoperative state, providing justification for surgery. Improvements could be observed for all symptoms. We discussed the importance of the sparing of the thalamus, the necessity to integrate the preoperative thalamocortical state as well as the relevance of the psycho-emotional dimension.

## Data Availability Statement

The datasets generated for this study are available on request to the corresponding author.

## Ethics Statement

All patients treated with this protocol signed an informed consent form after having been fully informed about the treatment, its results and risks. No ethical approval was sought because MRgFUS PTT has been approved by the swiss health state department and is covered by swiss social insurances.

## Author Contributions

MG: conception and design of the study, data acquisition and analysis, interpretation of the data, and co-drafted the manuscript. DJ: conception and design, data acquisition, interpretation of the data, and co-drafted the manuscript. DM and CF: co-drafted the manuscript. FR, AM, MS, RB, MK, PP, and CD: data acquisition. All authors read and approved the final manuscript.

### Conflict of Interest

MG, DM, FR, and DJ were employed by SoniModul Ltd., Center of Ultrasound Functional Neurosurgery, Solothurn, Switzerland. The Center for Ultrasound Functional Neurosurgery in Solothurn Switzerland is equipped with an Exablate Neuro of the Company Insightec, and patients are sent to receive interventions performed with this technology only. For DBS or gamma knife interventions patients are referred to and treated in other centers. The Center for Ultrasound Functional Neurosurgery in Solothurn and its employees did not receive any financial support by any medical company, including Insightec Ltd., for the whole time of this study. AM, MS, RB, MK, PP, and CD were employed by their medical offices or clinics, respectively. CF is supported by the Swiss National Science Foundation.

## Abbreviations

MTT: mammillothalamic tract
CEM: cumulative equivalent minutes at 43°C
MCL: mid-commissural line
MRgFUS: MR-guided focused ultrasound
PTT: pallidothalamic tractotomy
PD: Parkinson's disease
VLp Nucleus: Nucleus Ventralis Lateralis posterior
CMT: centrum medianum thalamotomy
UPDRS: Unified Parkinson's Disease Rating Scale
SDR: Skull density ratio.
